# Editorial: Cell therapy, liver diseases, and regeneration

**DOI:** 10.3389/fcell.2022.999305

**Published:** 2022-09-05

**Authors:** Wencheng Zhang, Guido Carpino, Kirk J. Wangensteen, Hexin Yan, Zhiying He

**Affiliations:** ^1^ Institute for Regenerative Medicine, Shanghai East Hospital, School of Life Sciences and Technology, Tongji University, Shanghai, China; ^2^ Shanghai Engineering Research Center of Stem Cells Translational Medicine, Shanghai, China.; ^3^ Shanghai Institute of Stem Cell Research and Clinical Translation, Shanghai, China.; ^4^ Department of Movement, Human and Health Sciences, University of Rome “Foro Italico”, Rome, Italy; ^5^ Department of Medicine, Division of Gastroenterology and Hepatology, Mayo Clinic, Rochester, MN, United States; ^6^ Renji Hospital Affiliated to Shanghai Jiaotong University, School of Medicine, Shanghai, China

**Keywords:** liver dieases, cell therapy, liver regeneration, animal models, cell tracking/imaging

The liver is the primary organ maintaining the homeostasis of nutrients and energy metabolism. It plays a role in deoxidation, glucose storage, secretory protein synthesis, and the production of bile required by the digestive system. Due to the complexity and abundance of its functions, the liver constantly is exposed to pathogenic factors that can lead to hepatitis, cirrhosis, and liver cancer, resulting in different forms of acute and chronic liver disease. Despite efforts to understand and treat liver diseases, when liver failure occurs, liver transplantation is still the most effective treatment. Cell-based therapy has become a potential alternative to liver transplantation. In experimental, preclinical, and in a few small clinical trials, hepatocytes and postnatal stem cells (including hepatic stem cells, hematopoietic stem cells, and mesenchymal stem cells) have been reported to effectively rescue or reduce liver injury. Yet, each of these cell or stem cell therapies has its own hopes and challenges, which require more research to become an effective treatment to help patients. In the Research Topic: *Cell Therapy, Liver Diseases, and Regeneration*, we aimed to gather a series of articles that address issues at the forefront of cell therapy and liver regeneration. Review articles and original research articles were selected for the liver injury models established to evaluate the functions of donor cells, cutting-edge imaging technology which enables tracking donor cells in the host liver in the single-cells manners with a course of time, and the mechanisms of liver cancers and liver regeneration which guide the translation of stem cell therapies from basic research to pre-clinical and clinical applications.

When it comes to cell therapy or stem cell therapy for liver disease, the quality and quantity of donor cells matter the most. Stem cells, whether pluripotent stem cells or multipotent stem cells, can be gradually induced into hepatocyte-like cells *in vitro* with precisely designed growth factor cocktails in a stepwise manner. The functional maturation of these hepatocyte-liked cells can be assessed by their gene expression patterns, functional protein secretion, and capacity for metabolism (i.e., cytochrome P450 activity). However, it is generally believed that *in vivo* evaluation of cell function has become the experimental gold standard. Due to the genetic and physiological similarities with humans and the capacity for genetic manipulation, mouse models have become the most favored and widely used model in the field of regenerative medicine. In the review by Du et al., key information on current mouse models simulating acute or chronic liver injuries was systematically summarized for studying the aging process and developing cell therapy strategies, and the “Five characters”, including, analogy, reproducibility, reversibility, adequate and appropriately body size to obtain adequate blood and tissue sample, were the suggested standards for an ideal animal model.

Properly designed mouse models are also suitable for studying liver diseases or cancers. In the study by Cheng et al., the authors established a mouse model of spontaneous HCC induced by oncogenes based on the insertion of transposon-mediated oncogene (AKT and NRASV12) into the genome of hepatocytes to induce tumorigenesis. Using this model, they found two novel HCC cell lines with distinct features of lipid metabolism related to cancer stemness and differential interplay with the immune system. This demonstrated that the syngeneic HCC mouse model is a practical tool for studying cancer stemness and discovering new therapies for liver cancers.

Of course, the immune system is involved in the pathogenesis of any form of liver diseases. Understanding the inflammatory responses under physiological and pathological states has attracted the attention of researchers who are focused on liver damaging and regeneration. In recent years, advanced techniques, including single-cell sequencing and spatial transcriptomics, have enabled researchers to better understand the relationship between liver inflammation and disease. However, these approaches only provide static and *ex vivo* information and do not allow the dynamic behavior and interaction patterns of immune cells in the inflammatory response to be evaluated in the unique anatomic structure and microenvironment of the liver. Wang and Wang reviewed exciting new findings from research that employs intravital microscopy (IVM), a powerful tool for directly observing cellular events within the intact organ of living animals. IVM can be applied to understand the immune composition and microenvironment in the healthy liver, as well as highly dynamic processes, such as the behavior of immune cells at a single-cell level during the process of inflammation. However, as they concluded at the end of the review, Wang and Wang pointed out that although IVM has been widely used to study the inflammatory response in the liver, most of these studies use artificial models that cannot fully reflect actual liver diseases. The combination of IVM with advanced spatial transcriptomic and proteogenomic techniques may make it possible to obtain comprehensive spatio-temporal information on inflammatory response and provide broad prospects for the diagnosis and treatment of liver disease.

Lastly, Yang et al. focused on summarizing and reviewing the progress, challenges, and prospects of mesenchymal stem cell (MSC) therapeutics for treating primary biliary cholangitis. Different from hepatocyte-related liver injuries, primary biliary cholangitis (PBC) is a cholestatic autoimmune liver disease, which is characterized by the gradual destruction of small intrahepatic bile ducts, eventually leading to cirrhosis, liver failure and even hepatocellular carcinoma. Although FDA-approved drugs are available, only some patients respond to treatment. And for patients with end-stage PBC, liver transplantation remains the only effective treatment. By reviewing basic research, preclinical research, and ongoing clinical trials, mesenchymal stem cells are shown to be a new option for treating PBC. By summarizing and discussing the current challenges and prospects of MSC-based therapy in clinical application, the authors hope to accelerate the application of MSCs to clinical practice settings, especially for refractory diseases such as PBC.

